# Medical History from the Earliest Times

**Published:** 1892-08-06

**Authors:** E. T. Withington


### Medical History from the Earliest Times.

By E. T. Withington, M.A., M.B. Oxon.

### XX.—GALEN.

According to the greatest of modern historians the second century of our era was the period in which mankind most nearly realised the fabled age of gold. The civilised world rested for a brief interval secure under the beneficent shadow of the Roman peace, and was ruled with paternal despotism by three great emperors, each of whom excelled his predecessor in wisdom and virtue. But if the age of Hadrian and the Antonines brought more happiness to mankind than that of Pericles, as periods in the history of human development they cannot for a moment be compared. Hippocrates, great as he was, might have found more than one equal among his contemporaries, but the second century can boast of but few men of genius, the most famous of whom was far inferior to Hippocrates. This was Claudius Galen (131-200), a man who not only knew all that there was to know in his age, but possessed sufficient talent and originality to acquire the position of a medical dictator, and to maintain it for more than a thousand years.

Galen was the most prolific of ancient authors; he wrote upon philosophy, mathematics, grammar, and law, as well as medicine, and his five hundred treatises equalled in number, and probably exceeded in bulk, those of Aristotle. Of the 181 which survive under his name about eighty are spurious or doubtful, or exist only in fragments and in Latin translations; but the remainder contain a complete and systematic view of Greek medicine, and gave laws to the civilised world in anatomy, physiology, and the doctrine and treatment of disease for upwards of fourteen centuries. Their influence can still be traced in many departments of modern medicine, and some of the teaching of the physician of Pergamus remains a possession for ever for the healing art. The following is a very brief outline of the more practical part of this teaching:—

Galen's anatomy was based partly on that of the Alexandrines and of his immediate predecessors Marinius and Rufus of Ephesus, but especially on his own dissections of monkeys and other animals, of which he mentions many kinds, from an elephant down to mice, birds, and fishes. Among other discoveries, he first pointed out the platysma, popliteus, and interossei muscles, the ductus arteriosus, and the three coats of the arteries. He omits no opportunity of asserting the great importance of anatomical knowledge in medicine, and declares that, though he rarely operated himself, he had often saved his colleagues by timely warnings from such disasters as befel a certain surgeon who divided the musculospiral, median, and ulnar nerves, and the brachial vessels owing to his ignorance of their position. The operator, says Galen, was so terrified that he only ligatured the vessels, and the partially-paralysed patient avenged himself by calling after him, "You cut my nerves."

Galen's physiology, though somewhat spoilt by excessive theorising, was no less admirable. He proved by experiment the falsity of Erasistratus' doctrine that the arteries contain air only, though at the same time he hindered the discovery of the circulation by three erroneous statements—(1) that the veins originate from the liver; (2) that the most important motion of the heart is its diastole; and (3) that the septum between the ventricles is permeable. In opposition to his predecessors, he declared that respiration serves not to cool the body, but to maintain the animal heat, and made the happy suggestion that when we discover what part of the atmosphere supports combustion we shall also know what is the source of the bodily temperature. His comparison of sound to a wave-like movement was equally fortunate.

But Galen's chief physiological work was his investigation of the nervous system, in which he made extensive use of vivisections. He distinguished sensory, motor, and mixed nerve trunks, traced the connection between the vagus and the sympathetic, showed the importance of the recurrent nerves for the production of voice, and, above all, pointed out that the nerves have no power in themselves, but merely conduct impulses to and from the brain and spinal cord. This knowledge gained him a victory over the hated Methodists. Pausanias, a celebrated sophist, had complained of loss of sensation in the fourth and fifth fingers of his left hand, and the Methodist physicians who attended him, considering the disease to be due to "constriction," applied poultices locally, but without effect. Galen was then summoned, and at once recognised the distribution of the ulnar nerve. On inquiry he found that the sophist had been thrown from his chariot some time before and had struck his back against a stone. He thereupon applied counter-irritants to the region of origin of the brachial plexus, and the patient recovered, to the confusion of the Methodists and the triumph of Galen, who tells the story at least three times in his extant writings.

Medicine, according to Galen, rests upon anatomy and physiology, and is the art of maintaining and restoring health. Disease is the opposite of health, and, taking the simplest of his many definitions, is "an abnormal affection of the body giving rise to a lesion of function." He further divides diseases into three classes—(1) those affecting the "similar parts," or simple tissues, muscle, nerve, ligaments, &c.; (2) those of the compound tissues, or organs, heart, lungs, &c.; (3) those affecting the body generally, and, especially, the four humours. Diseases of this last class are "dyscrasiæ," and contrast with the complete and harmonious mixture of the humours "eucrasia." But this state of ideal health rarely occurs, and the predominance of the various humours gives rise to the "temperaments," sanguine, phlegmatic, bilious, or melancholic, which, though partaking of the nature of disease, are not actually to be so called unless they produce a perversion of function.

Galen's definitions of the causes, symptoms, and signs of disease correspond in their broader outlines with those still accepted, but he so delighted in defining and classifying, and carried out those processes with such subtlety and minuteness that he defeated his own purpose, and diverted the minds of generations of disciples from the more practical part of his teaching.

Our short remaining space will be devoted to his therapeutic doctrines, which display at once the best and worst sides of the dogmatic school. As an example of the former, we may take his doctrine of the "indications," under which term he comprises "whatever enables us to draw conclusions as to treatment apart from experience." The indications formed the touchstone of distinction between the three great medical sects. The Empirics rejected them entirely, the Methodists reduced them to one only—the restoration of the normal state of the pores by the use of contraries, while to the Dogmatists they formed the basis of all rational treatment. The first and greatest indication is to remove the cause of the disease or to prevent its action; a second class arises from the symptoms, any of which may form ground for treatment—if against Nature, by contraries, if in accordance with Nature, by similars. Other sources of indications are—the temperament of the patient, the season of the year and external circumstances generally, and, finally, as Galen curiously adds, the patient's dreams.

His teaching as to the action of drugs was less excellent, but, unfortunately, far more influential. He held that some, such as emetics, purgatives, poisons and their antidotes, act through their whole substance, and are, in a sense, specifics, a doctrine consonant with the older dogmatic theory of the specific action of purgatives, which still survives in the modern terms hydrogogue and cholagogue. But most drugs act, according to Galen, through one of the elementary qualities—heat, cold, dryness, and moisture, which they possess not in an actual but a potential form, each quality being further divisible into four degrees, according to its intensity. Thus pepper and opium are (potentially) hot and cold respectively in the fourth degree. This theory, after being developed to an absurd extent by the mediæval physicians, has happily vanished, leaving us only the terms "actual" and "potential" as applied to cauteries.

It is a curious fact that the treatise "On Ancient Medicine," in which Hippocrates mentions this doctrine and rejects it, is the only one of his genuine works on which Galen has not written a commentary, probably because he felt he could not reconcile it with his own teaching.

